# TM5441, a plasminogen activator inhibitor-1 inhibitor, protects against high fat diet-induced non-alcoholic fatty liver disease

**DOI:** 10.18632/oncotarget.21120

**Published:** 2017-09-21

**Authors:** Seon Myeong Lee, Debra Dorotea, Inji Jung, Tetsuo Nakabayashi, Toshio Miyata, Hunjoo Ha

**Affiliations:** ^1^ Graduate School of Pharmaceutical Sciences, College of Pharmacy, Ewha Womans University, Seoul, Republic of Korea; ^2^ United Centers for Advanced Research and Translational Medicine, Tohoku University Graduate School of Medicine, Miyagi, Japan

**Keywords:** plasminogen activator inhibitor 1, non-alcoholic fatty liver disease, high-fat diet, insulin resistance, organelle biogenesis

## Abstract

Recent evidences showed that elevation of plasminogen activator inhibitor 1 (PAI-1) was responsible in mediating obesity-induced non-alcoholic fatty liver disease (NAFLD) and metabolic disorders. Here, we investigated the effect of TM5441, an oral PAI-1 inhibitor that lacks of bleeding risk, on high-fat diet (HFD)-induced NAFLD. HFD-fed C57BL/6J mice was daily treated with 20 mg/kg TM5441. To examine the preventive effect, 10-week-treatment was started along with initiation of HFD; alternatively, 4-week-treatment was started in mice with glucose intolerance in the interventional strategy. *In vivo* study showed that early and delayed treatment decreased hepatic steatosis. Particularly, early treatment prevented the progression of hepatic inflammation and fibrosis in HFD mice. Interestingly, both strategies abrogated hepatic insulin resistance and mitochondrial dysfunction, presented by enhanced p-Akt and p-GSK3β, reduced p-JNK signaling, along with p-AMPK and PGC-1α activation. Consistently, TM5441 treatment in the presence of either PAI-1 exposure or TNF-α stimulated-PAI-1 activity showed a restoration of mitochondrial biogenesis related genes expression on HepG2 cells. Thus, improvement of insulin sensitivity and mitochondrial function was imperative to partially explain the therapeutic effects of TM5441, a novel agent targeting HFD-induced NAFLD.

## INTRODUCTION

Non-alcoholic fatty liver disease (NAFLD) can range from simple steatosis, inflammatory steatohepatitis (NASH) with increasing severity of fibrosis, and ultimately cirrhosis [1]. The prevalence of NAFLD has risen steadily in parallel with the burgeoning number of obesity and diabetes patients. Therefore, NAFLD represents the most common cause of liver disease in developed countries [2, 3]. However, detailed pathogenic mechanism of NAFLD progression is not yet fully understood which limits the development of therapeutic agents [4, 5].

Plasminogen activator inhibitor (PAI)-1 is a 50 kDa single-chain glycoprotein and member of serine protease inhibitors. PAI-1 is widely known as an endogenous inhibitor of plasminogen activation by tissue-type and urokinase-type plasminogen activator (t-PA and u-PA, respectively) [6]. Plasma and tissue concentration of PAI-1 are extremely low on basal condition but elevated under pathological conditions. Increased PAI-1 activity has been associated with higher risk of metabolic syndromes, cardiovascular events, and tissue fibrosis [7, 8].

Growing evidences coherently imply that PAI-1 mediates the development of hepatic steatosis in metabolic disorder condition. Clinically, a steady elevation of plasma and hepatic PAI-1 levels was correlated with the progression of NAFLD [9]. Animal studies, with either genetic- or diet-induced obesity, showed that PAI-1 activation mediated obesity-induced insulin resistance [10–14]. In those studies, PAI-1 null mice improved glucose intolerance, insulin resistance, hyperlipidemia [11–13] and protected against hepatic steatosis [12, 14]. Additionally, PAI-1 knockout or knockdown in bile duct ligation (BDL) induced-mice might attenuate hepatic fibrosis [15–17]. Overall, those studies suggest that a complete PAI-1 deficiency alleviates a range of disorders found in NAFLD. However, no study has elucidated the therapeutic effect of pharmacological PAI-1 inhibition on liver injury.

Therefore, the present study investigated the efficacy of TM5441, a novel and orally active small molecule inhibitor of PAI-1, in ameliorating high fat diet (HFD)-induced NAFLD. TM5441 exceptionally lacks of bleeding risk and specifically inhibits PAI-1, no inhibitory effect was observed on other serine proteases, such as anti-thrombin III and α2-antiplasmin [18–20]. Numerous studies has demonstrated the protective effect of TM5441 and its analogue on various pathological conditions, such as hypertension [20], inflammation- and diabetic- induced kidney injury [21, 22], lung fibrosis [23], and cellular senescence [24, 25].

In response to those results, our previous study also showed the protective effect of TM5541 in HFD-induced obesity and adipocyte injury by maintaining mitochondrial function [26]. However, the role of PAI-1 in mitochondria remains controversial. PAI-1 overexpression in cancer-associated fibroblasts enhanced mitochondrial biogenesis in adjacent breast cancer cell which promotes metastasis [27]. Hence, investigating mechanism of PAI-1 inhibition on ameliorating HFD-induced NAFLD can be imperative to provide another insight of the role of PAI-1 in mitochondrial biogenesis.

The present study was specifically directed to investigate the effect of either early or delayed treatment of TM5441 in the HFD-induced NAFLD. Emphasizing on its capacity in remodeling insulin signaling and mitochondrial fitness, we examined the efficacy of TM5441 in decreasing hepatic steatosis, inflammation, and ultimately fibrosis, which are the hallmarks of NAFLD progression.

## RESULTS

### Early TM5441 treatment prevented HFD-induced hepatic steatosis

**Figure 1:**
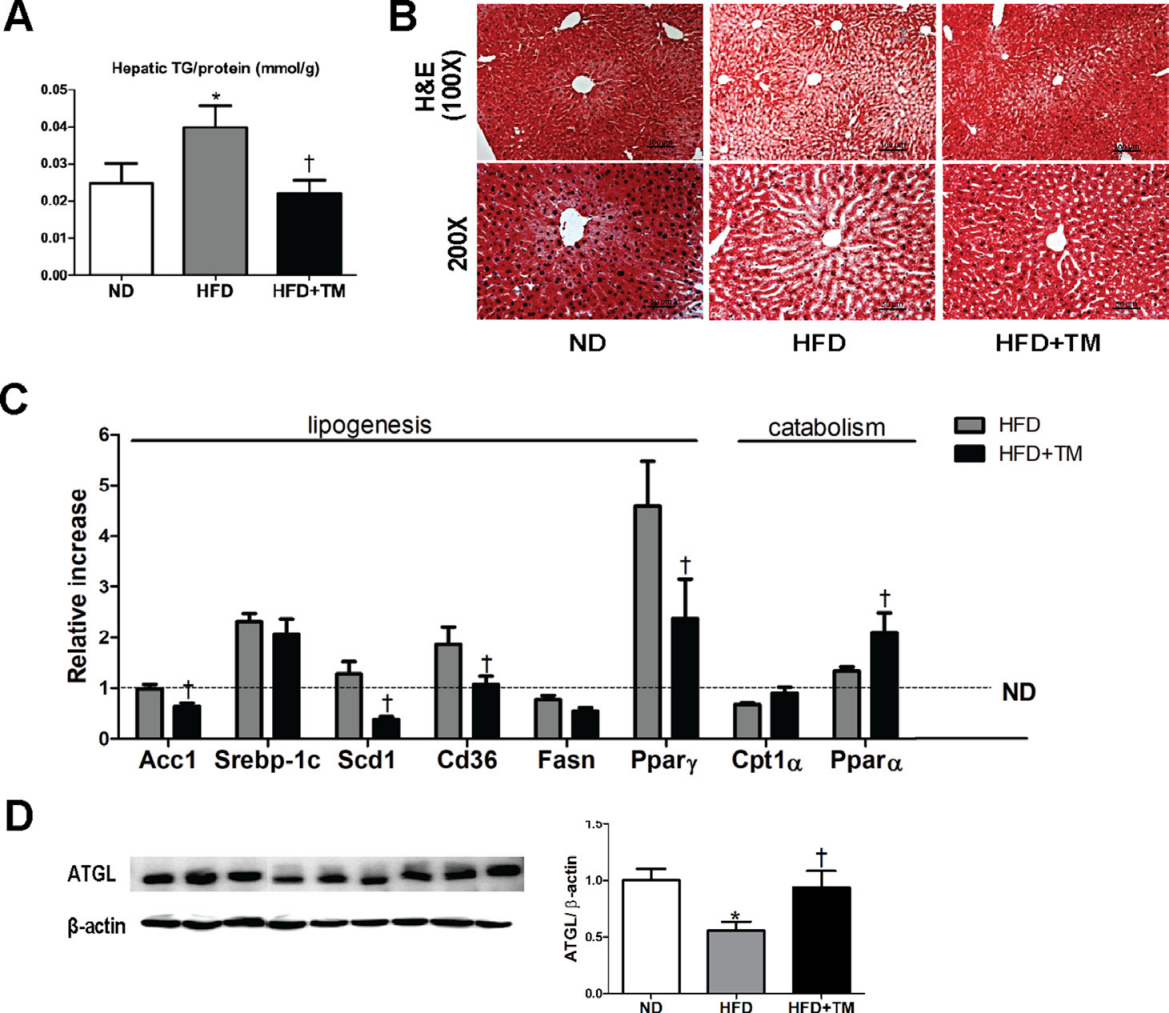
Early TM5441 treatment prevents HFD-induced hepatic steatosis After 10-week-HFD, mice were sacrificed and hepatic tissues were analyzed. (**A**) Hepatic TG content/liver protein (mmol/g) was measured. (**B**) 5 μm liver section was stained with H&E. The scale bar indicates 100 μm and 50 μm for 100× and 200× magnification, respectively. (**C**) mRNA expression levels of genes representing lipogenesis and lipolysis were quantified with RT-qPCR analysis. (**D**) Protein lysate of the liver was prepared for immunoblot analysis of ATGL. Data are shown as mean ± SE of 7–8 mice. *p < 0.05 vs ND, ^†^*p* < 0.05 vs HFD.

Body weight and metabolic parameters of experimental animal in the prevention regimen were as described in our previous publication [26]. We showed that compared to ND mice, 10-week-HFD mice had significant elevations of hepatic TG content and lipid accumulation in liver tissue which were remarkably reduced in response to TM5441 (Figure 1A, 1B). Lipogenesis-related genes were also evaluated; Acc1, Scd1, Cd36, and PPARγ were significantly decreased in TM5441-treated mice (Figure 1C). While, lipid catabolism was significantly increased in response to TM5441 treatment as shown by elevations of PPARα mRNA expression (Figure 1C) and ATGL protein level (Figure 1D).

### Early TM5441 treatment prevented hepatic insulin resistance in HFD-fed mice

**Figure 2:**
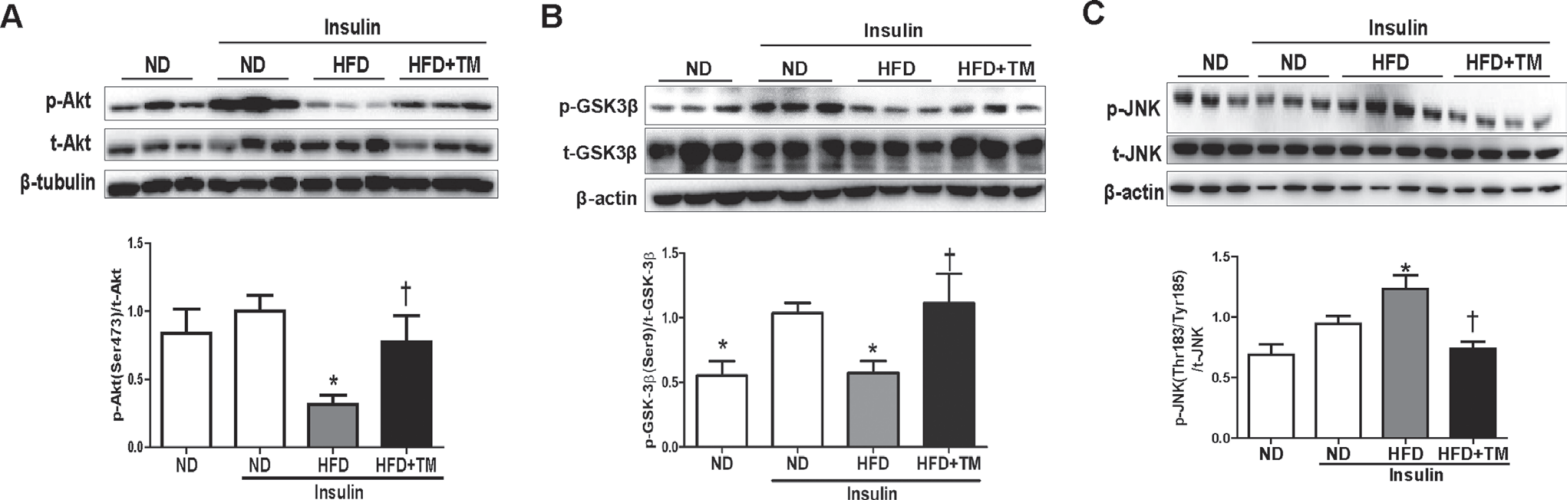
Early TM5441 treatment enhanced hepatic insulin signaling in HFD mice During sacrifice, liver were taken before and after insulin injection. Then, protein lysate of the liver was prepared for immunoblot analysis of (**A**) Akt, (**B**) GSK-3β, and (**C**) JNK in both total and phosphorylated forms. Expression level of phosphorylated and total form quantified by densitometer and normalized with β-tubulin or β-actin as indicated. Data are shown as mean ± SE of 7–8 mice. *p < 0.05 vs ND, ^†^*p* < 0.05 vs HFD.

To investigate whether HFD-induced hepatic insulin resistance might be prevented by TM5441, we measured proteins involved in insulin signaling, such as Akt, GSK-3β, and JNK. Akt and GSK-3β phosphorylation were significantly decreased in HFD mice compared to ND mice, which were reversed in response to TM5441 (Figure 2A, 2B). Then, the elevated level of p-JNK in HFD mice was effectively reduced by TM5441 treatment (Figure 2C).

### Early TM5441 treatment prevented HFD-induced hepatic inflammation

**Figure 3:**
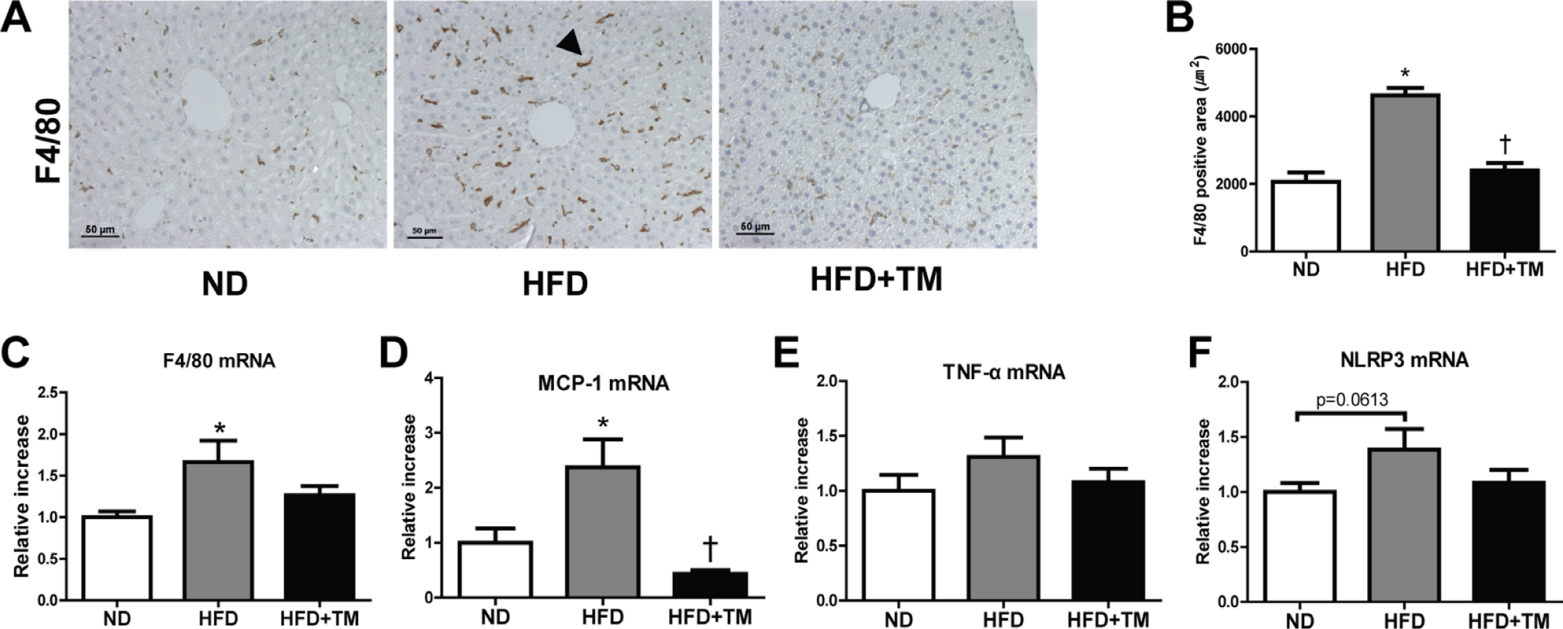
Early TM5441 treatment suppressed hepatic inflammation in HFD mice (**A**, **B**) Anti-inflammatory effect of TM5441 was analyzed through quantifying positive F4/80 IHC staining area in the liver section. Magnification, 200×; scale bar, 50 μm. Pro-inflammatory mRNA expressions in the liver, such as (**C**) F4/80, (**D**) MCP-1 (**E**) TNF-α, (**F**) NLRP3, were measured by RT-qPCR. Data are shown as mean ± SE of 7–8 mice. *p < 0.05 vs ND, ^†^*p* < 0.05 vs HFD.

To determine anti-inflammatory effect of TM5441 in HFD mice, we evaluated the expression of pro-inflammatory markers in the liver. Positive F4/80 IHC staining, along with mRNA expression of F4/80 and MCP-1 were significantly increased in HFD mice and reduced by TM5441 treatment (Figure 3A–3D). In spite of less statistical significance, TNF-α and NLRP3 showed similar increment trend in HFD mice, followed by reduction upon TM5441 treatment (Figure 3E, 3F).

### Early TM5441 treatment protected against HFD-induced hepatic fibrosis

**Figure 4:**
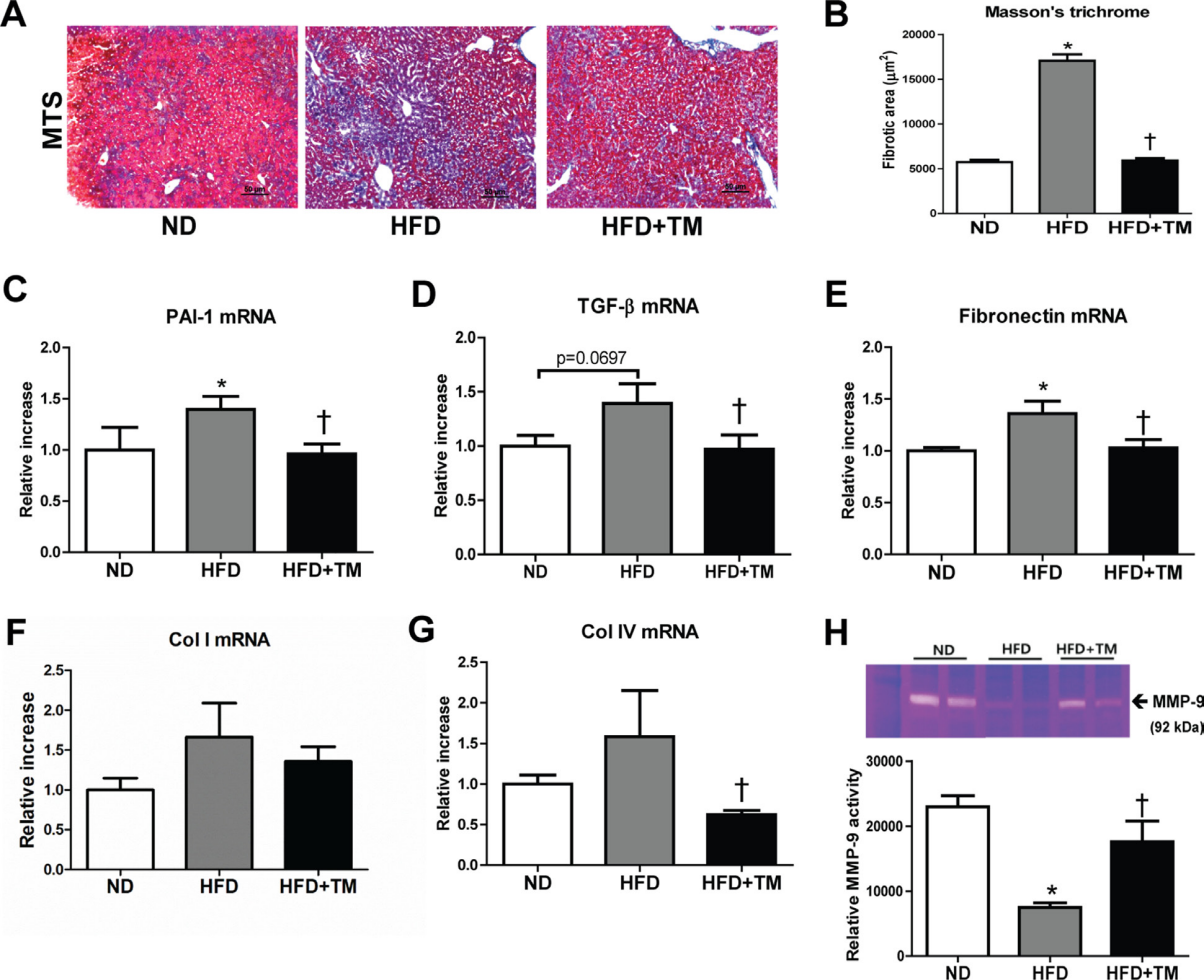
Early TM5441 treatment attenuates hepatic fibrosis in HFD mice (**A**, **B**) Collagen accumulation in the liver was detected with Masson's trichrome staining. Magnification, 200x; scale bar, 50 μm. Liver mRNA expressions of (**C**) PAI-1, (**D**) TGF-β1, (**E**) fibronectin, (**F**) collagen Ia1 (Col I), (**G**) collagen IVa1 (Col IV) were measured by RT-qPCR. (**H**) The activity of MMP-9, factor involved in ECM degradation, was measured with gelatin zymography. Data are shown as mean ± SE of 7-8 mice. *p < 0.05 vs ND, ^†^*p* < 0.05 vs HFD.

We further investigated the preventive effect of TM5441 on extracellular matrix (ECM) accumulation and fibrosis, the hallmarks of NAFLD progression. First, we confirmed the ECM accumulation in the liver of HFD mice presented by positive collagen area in Masson's trichrome stained section. Remarkably, this accumulation was reduced by TM5441 treatment (Figure 4A, 4B). The anti-fibrotic effect was further supported by decreased mRNA levels of fibrogenic genes, such as PAI-1, TGF-β1, fibronectin, and collagen IV in TM5441-treated HFD mice (Figure 4C–4G). Finally, gelatin zymography of liver lysate revealed that TM5441 significantly induced MMP-9 activation in HFD mice (Figure 4H), partially explaining the anti-fibrotic mechanism of TM5441.

### Early TM5441 treatment improved AMPK and PGC-1α activity in HFD-fed mice

**Figure 5:**
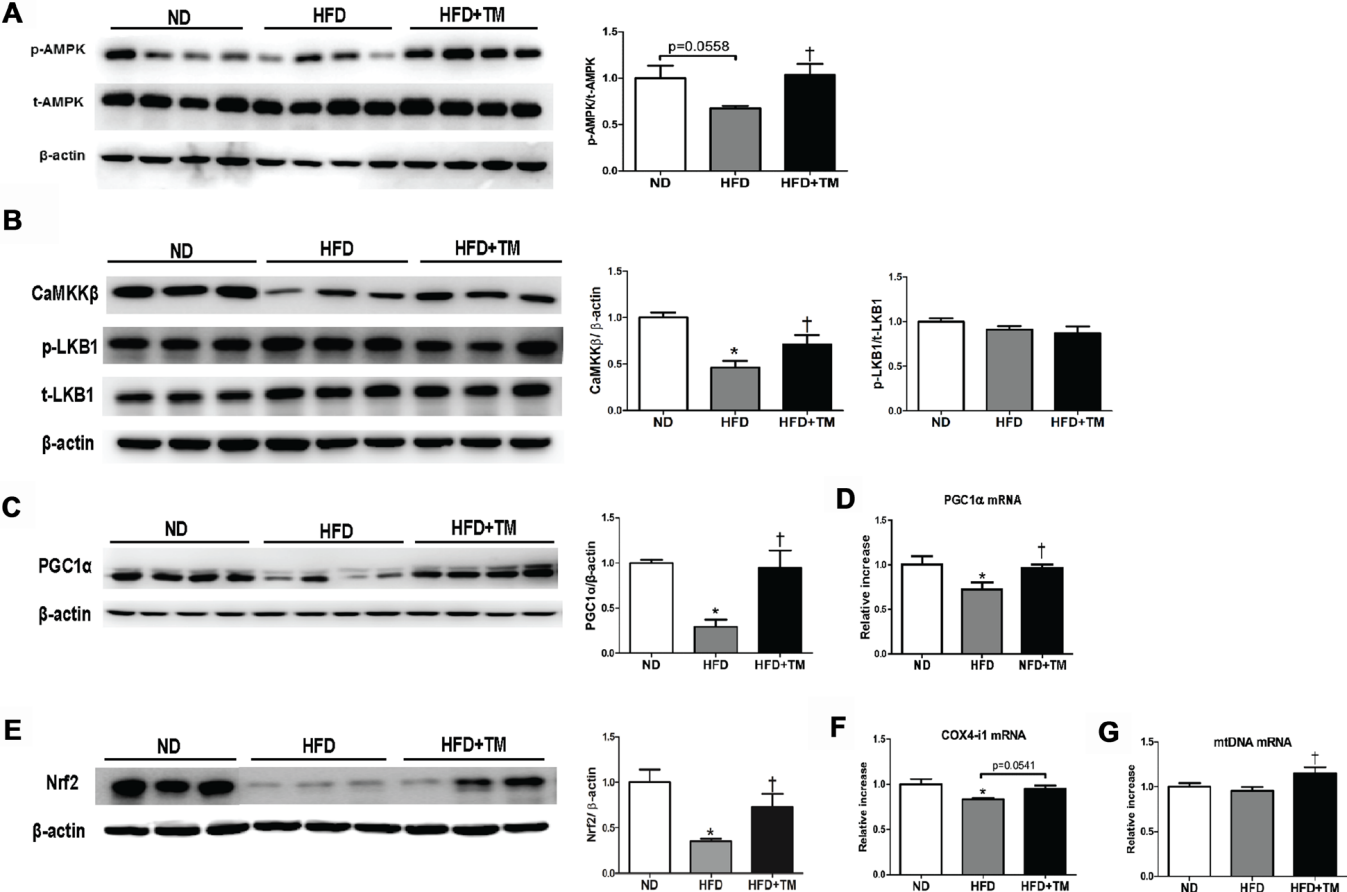
Early TM5441 treatment activates p-AMPK and PGC-1α in HFD mice Liver protein expressions of (**A**) total- and phospho- AMPK along with its upstream, including (**B**) CaMKKβ, p-LKB1, and t-LKB1 were analyzed. Then, we measured the (**C**) protein and (**D**) mRNA expression of PGC-1α. (**E**) Protein expression of Nrf2, the transcription factor related to PGC-1α, and mRNA expression of genes related to mitochondrial biogenesis, such as (**F**) COX4-i1, and (**G**) mtDNA were all measured. Data are shown as mean ± SE of 7–8 mice. *p < 0.05 vs ND, ^†^*p* < 0.05 vs HFD.

For a further insight about how TM5441 halts the deterioration induced by high fat feeding, we evaluated its efficacy in modulating AMPK, a regulator of energy metabolic homeostasis [28], as well as PGC-1α, a master regulator of mitochondrial biogenesis [29]. AMPK activation was reduced in HFD mice and reversed upon TM5441 treatment (Figure 5A). Regarding the AMPK upstream, CaMKKβ was decreased in HFD and elevated in response to TM5441 treatment. On the other hand, the activity of LKB1 was not affected in response to either HFD or TM5441 treatment (Figure 5B). Then, mRNA and protein levels of PGC-1α were decreased in HFD mice and brought to normal levels by TM5441 treatment (Figure 5C, 5D). Nrf2, a transcription factor interacted with PGC-1α, was also normalized in the treatment group (Figure 5E). Other PGC-1α target genes were also measured. COX4-i mRNA expressions in HFD-fed mice were increased in response to TM5441 (Figure 5F). In spite of constant level of mtDNA following HFD, its mRNA expression was significantly increased in response to TM5441 treatment compared to both ND and HFD (Figure 5G).

### Delayed TM5441 treatment ameliorated HFD-induced NAFLD and metabolic disorders

**Figure 6:**
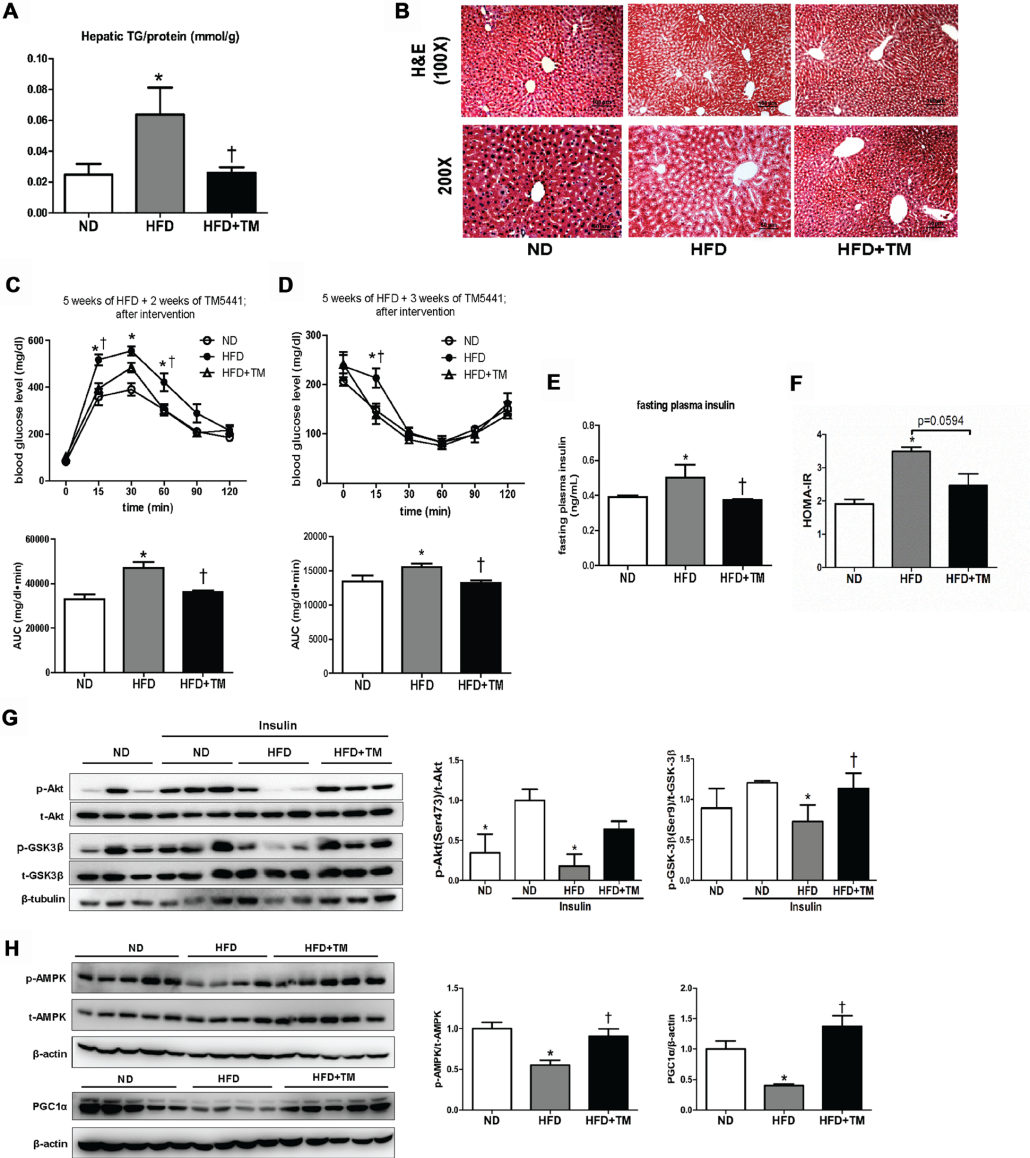
Delayed TM5441 treatment ameliorated HFD-induced NAFLD and metabolic disorder 4-week-interventional treatment was initiated on 10-week-old HFD mice with glucose intolerance. (**A**) Hepatic TG content/liver protein (mmol/g) was measured. (**B)** H&E staining depicted lipid accumulation in the liver. The black scale bar indicates 100 μm and 50 μm for 100× and 200× magnification, respectively. (**C**) GTT and (**D**) ITT measurement were performed after 2 weeks and 3 weeks of TM5441 treatment, respectively. AUC of blood glucose were then calculated and analyzed. (**E**) Fasting plasma insulin levels were measured and (**F**) HOMA-IR were subsequently calculated. (**G**) Immunoblot clarified insulin signaling activity presented through detection of Akt and GSK3β in the absence and presence of insulin stimulation. (**H**) Then, AMPK phosphorylation and PGC-1α were analyzed to see the activation of mitochondrial biogenesis. Data are shown as mean ± SE of 7–8 mice. *p < 0.05 vs ND, ^†^*p* < 0.05 vs HFD.

Besides prevention effects, we tested whether TM5441 treatment showed therapeutic benefit in HFD-fed mice that had developed metabolic disorders, presented by glucose intolerance state (Supplementary Figure 1A, 1B). TM5441 treatment attenuated HFD-induced hepatic TG content and lipid accumulation on hematoxylin and eosin (H&E)-stained liver section (Figure 6A, 6B). Decreased plasma FFA and TG were also observed in HFD mice treated with TM5441 (Supplementary Figure 2A, 2B). In contrast, high plasma level of PAI-1 in HFD mice was not adequately decreased after 4-week-TM5441 treatment (Supplementary Figure 2C).

Then, we verified that delayed TM5441 treatment improved insulin sensitivity in HFD mice. First, GTT and ITT results suggested that after 2–3 weeks of TM5441 treatment, glucose and insulin tolerance were significantly improved in HFD mice (Figure 6C, 6D). Additionally, fasting plasma insulin level and HOMA-IR index of HFD mice were significantly reduced by TM5441 treatment (Figure 6E, 6F). Persistently, immunoblot results indicated increased Akt and GSK-3β phosphorylation, suggesting improved insulin signaling in response to TM5541 (Figure 6G). Finally, the effect of TM5441 on hepatic mitochondrial biogenesis of HFD-fed mice were also elucidated. Decreased p-AMPK and PGC-1α were observed in HFD mice which were reversed by TM5441 treatment (Figure 6H).

### PAI-1 siRNA attenuated TNF-α-induced mitochondrial dysfunction in HepG2 cells

**Figure 7:**
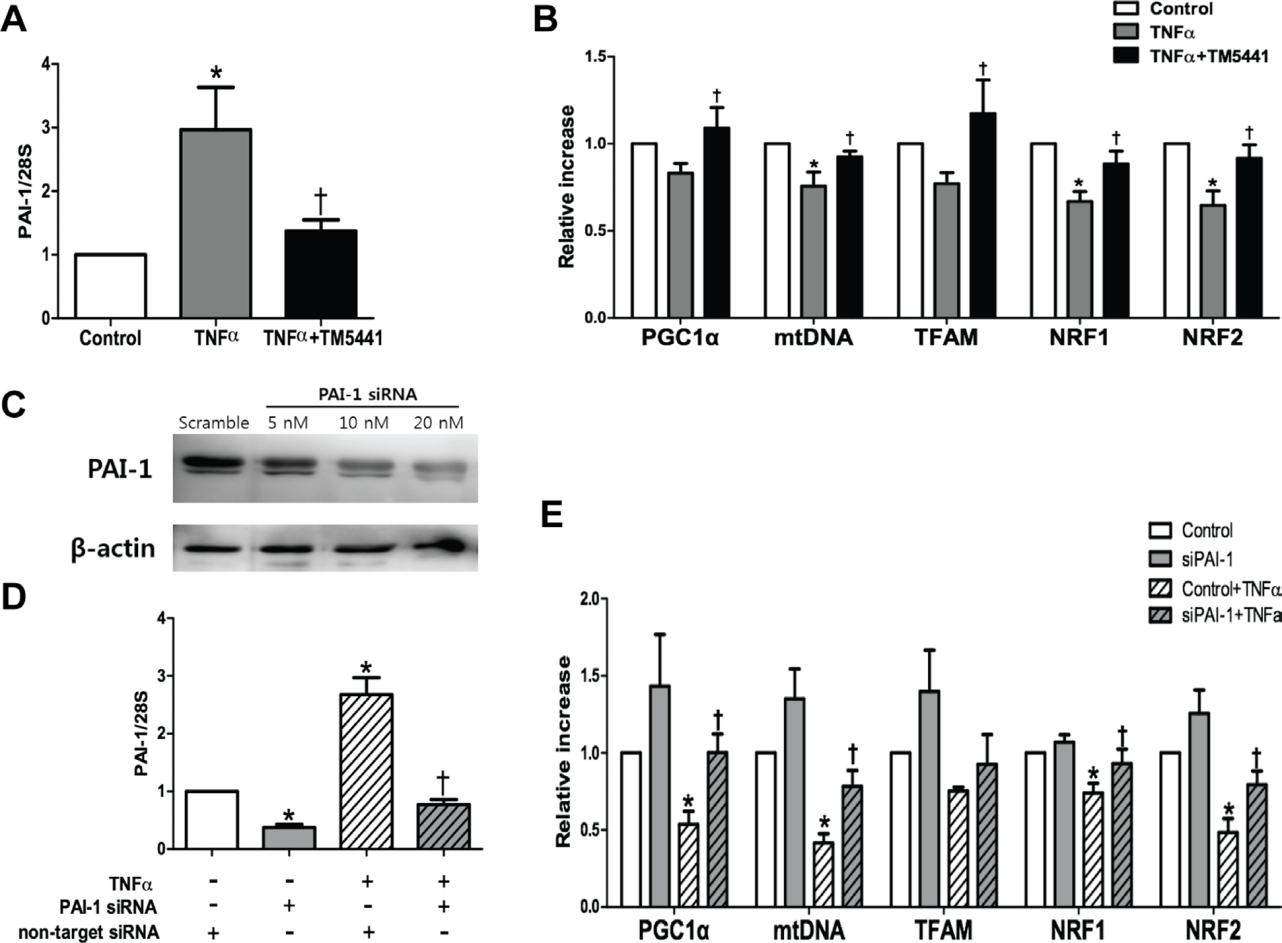
TM5441 pre-treatment and siPAI-1 improved TNF-α-induced mitochondrial dysfunction in HepG2 cells HepG2 cells were pretreated with 20 μM TM5441 before the exposure of 50 ng/ml TNF-α for 24 hours. mRNA expressions of (**A**) PAI-1 and (**B)** mitochondria biogenesis-related genes, such as PGC-1α, mtDNA, TFAM, NRF1, and NRF2 were measured by RT-qPCR. Then, PAI-1 is downregulated with siRNA in which confirmed by immunoblot analysis. (**C**) 20 nM siPAI-1 was transfected 24 hours before the exposure of 50 ng/ml TNF-α for 24 hour. The mRNA gene expression of (**D**) PAI-1 and (**E**) the mitochondrial function-related genes were measured by RT-qPCR. Data are presented as mean±SE of 4-5 experiments. *p < 0.05 vs. control, ^†^*p* < 0.05 vs. control+TNF-α.

To examine the putative effect of PAI-1 in the hepatic mitochondrial function, the HepG2 cells was exposed to TNF-α, a pivotal pro-inflammatory marker and one of the strongest PAI-1 activators [30]. TM5441 pre-treatment downregulated PAI-1 mRNA expression (Figure 7A) and upregulated mitochondrial biogenesis-related genes, such as PGC-1α, mtDNA, TFAM, NRF1, and NRF2 (Figure 7B). This result was further confirmed by silencing PAI-1 with siRNA in the presence or absence of TNF-α exposure (Figure 7C, 7D). In the basal condition, PAI-1 knockdown caused insignificant elevation of mitochondrial biogenesis-related genes. However, PAI-1 knockdown in the presence of TNF-α stimuli significantly increased PGC-1α, mtDNA, NRF1, and NRF2 mRNA level (Figure 7E).

### TM5441 inhibited PAI-1-induced mitochondrial dysfunction and lipid accumulation in HepG2 cells

**Figure 8:**
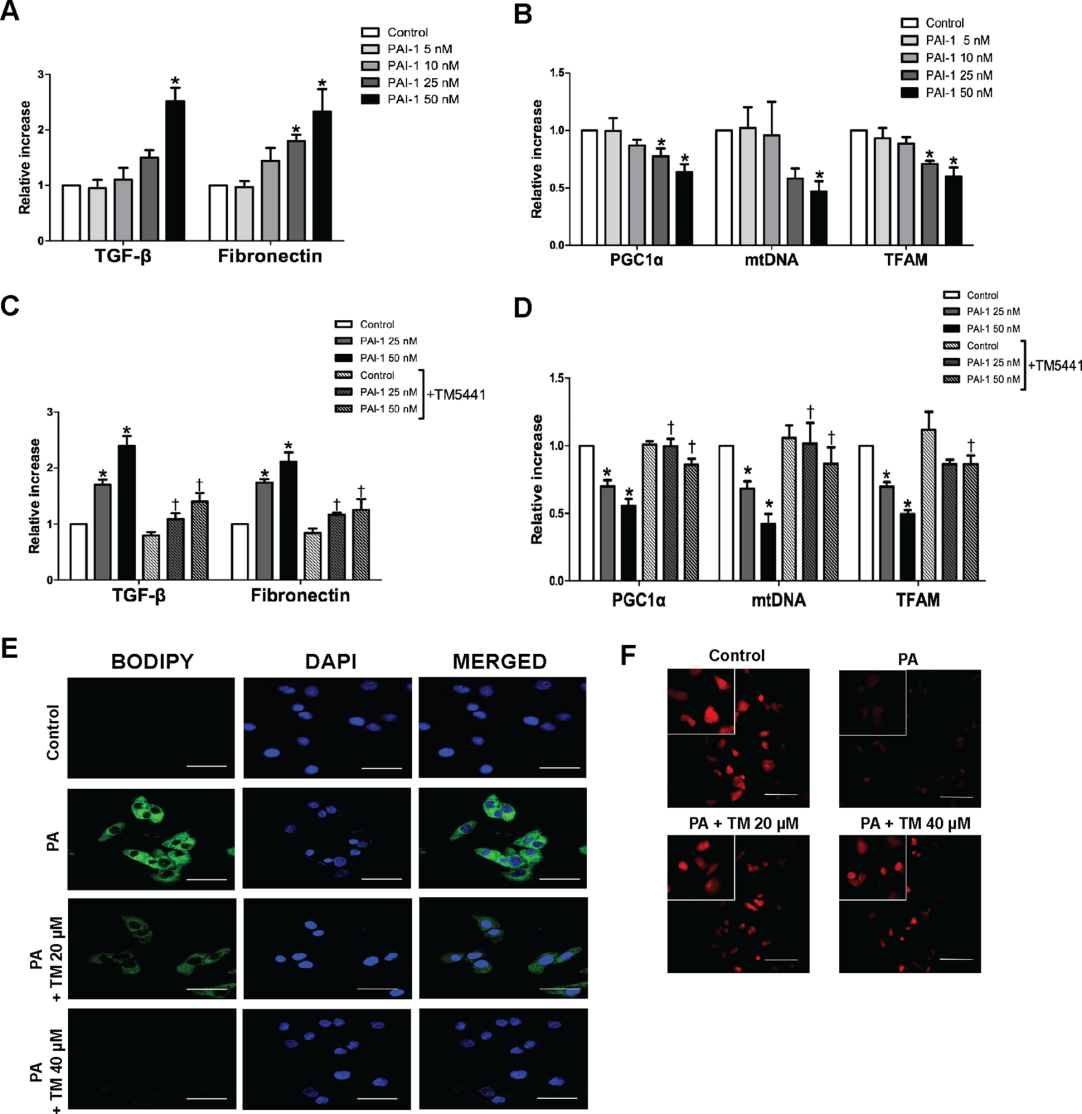
TM5441 inhibited downregulation of mitochondrial biogenesis-related genes and lipid accumulation in HepG2 cells HepG2 cells were stimulated with PAI-1 recombinant for 12 hours, followed by measurement of (**A**) TGF-β, fibronectin, (**B**) PGC-1α, mtDNA, and TFAM mRNA expressions. Then, HepG2 cells were pre-treated with 20 μM TM5441 prior to 12-hour-PAI-1 exposure (25 or 50 nM). The direct effect of TM5441 on mRNA expressions of (**C**) TGF-β and fibronectin, (**D**) PGC-1α, mtDNA and TFAM were further analyzed. While, HepG2 cells was pre-treated with TM5441 (20 or 40 μM) prior to 24-hour-PA stimulation. (**E**) Neutral lipid accumulation was detected by BODIPY staining and (**F**) mitochondria was labeled with Mitotracker^TM^ staining. Scale bar indicates 50 μm. Data are presented as mean ± SE of 4 experiments. *p < 0.05 vs. control, ^†^*p* < 0.05 vs. response to the same concentration of PAI-1 in the absence of TM5441.

Since TNF-α-induced mitochondrial dysfunction was reversed by PAI-1 knockdown, we investigated whether PAI-1 directly inhibited mitochondrial biogenesis. HepG2 cells treated with PAI-1 recombinant, especially 50 nM, showed upregulation of TGF-β1 and fibronectin mRNA level (Figure 8A), as well as downregulation of mitochondrial biogenesis genes, such as PGC-1α, mtDNA, and TFAM (Figure 8B). Accordingly, TM5441 pre-treatment under 50 nM PAI-1 exposure, significantly reduced TGF-β and fibronectin mRNA level (Figure 8C) as well as upregulated PGC-1α, mtDNA, and TFAM mRNA expressions, compared to identical concentration of PAI-1 in the absence of TM5441 (Figure 8D).

Since activation of PGC-1α is associated with improvement of mitochondrial function, we confirmed whether TM5441 treatment can directly reduce excessive lipid accumulation and increase mitochondrial biogenesis in HepG2 cells. TM5441 pre-treatment significantly suppressed lipid droplet accumulation on HepG2 cells stimulated with PA, shown by less intensity of BODIPY staining (Figure 8E). Then, mitochondrial biogenesis was accentuated by increased of mitochondria labeled by Mitotracker^TM^ staining upon TM5441 pre-treatment (Figure 8F).

## DISCUSSION

**Figure 9:**
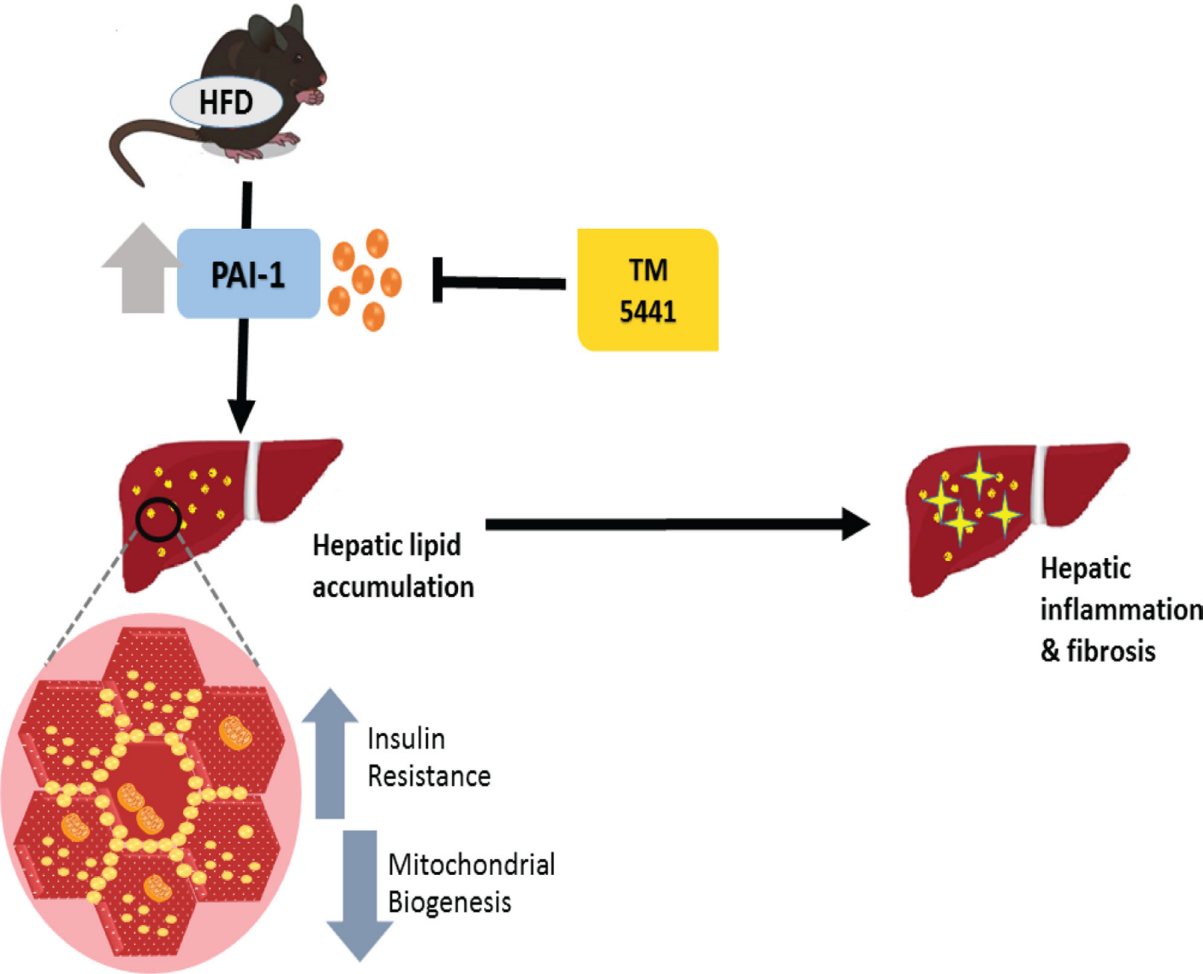
A schematic diagram showing the inhibition of PAI-1 by TM5441 ameliorates NAFLD partially through increasing mitochondrial biogenesis

Abundant results have demonstrated that complete PAI-1 deficiency protected against diet-induced insulin resistance and hepatic steatosis [11–14]. Interestingly, our present study is the first to establish the therapeutic effect of TM5441, a PAI-1 inhibitor, on HFD-induced NAFLD. Both early and delayed TM5441 treatment decreased hepatic triglyceride content and lipid accumulation. In particular, early treatment prevented the development of hepatic inflammation and fibrosis in HFD mice. Interestingly, deteriorated insulin signaling and mitochondrial biogenesis involved in the disease progression were remarkably reversed by both treatment strategies (Figure 9).

The development and progression of NAFLD represents a complex pathophysiology [31]. In the present study, we used HFD-fed mice to induce NAFLD. Although previous studies with HFD-fed rodents showed less consistency in progressing through liver fibrosis, this model can replicate metabolic parameters alteration as found in human NAFLD, such as obesity, hyperinsulinemia, and insulin resistance [32].

In this study, 10-week-HFD presented hepatic steatosis, inflammation, and eventually fibrosis. As stated in previous publication, the metabolic parameters exhibited body-weight gain, insulin resistance, and higher serum PAI-1 [26]. The notion of insulin resistance was clearly presented by increased p-JNK along with reduced p-Akt and p-GSK3β in HFD mice. In the steatohepatitis, pro-inflammatory mediators activates JNK signaling, leading to insulin resistance [33]. Intricately, hepatic insulin resistance augments steatosis through increased de novo lipogenesis, decreased FFA oxidation, decreased very low-density-lipoprotein secretion, and increased FFA efflux due to increased adipose tissue's lipolysis [31].

Persistently, our study suggested an elevation of hepatic TG composition and lipid accumulation, as well as higher serum FFA and TG in HFD mice. Then, HFD obviously caused an excessive lipogenesis with a lower lipolysis rate. Early TM5441 treatment significantly downregulated lipogenesis-related genes (Acc1, Scd1, Cd36, and PPARγ) and upregulated lipolysis-related genes (PPARα). Moreover, treated mice also exhibited an increase in ATGL, a major hepatic lipase regulating TG turnover [34, 35]. Hepatic steatosis was attenuated by TM5441 along with improvement of hepatic insulin sensitivity presented by decreased p-JNK along with increased p-Akt and p-GSK3β. Moreover, delayed treatment might also bring the GTT, ITT, and fasting plasma insulin to the normal level as observed in ND mice.

The strong correlation between insulin sensitivity and intracellular TG content (i.e. liver and muscle tissues) has been well-established in human and animal studies of obesity-related insulin resistance and type 2 diabetes [36, 37]. Lower TG content and lipid accumulation in liver in response to TM5441 is potentially a protective mechanism contributing to the improvement insulin sensitivity. This protection effect is supported by the result in HFD-fed PAI-1^-/-^ mice. PAI-1 deficiency was related to lower hepatic TG and protection from obesity and insulin resistance. In PAI-1^-/-^ mice, increased skeletal muscle UCP-2 and UCP-3 may contribute to increased metabolic rates and energy expenditure, leading to protection against insulin resistance [12].

Moreover, inflammation and insulin resistance in adipose tissue during obesity can potentiate hepatic inflammation, insulin resistance, and de novo lipogenesis that lead to steatosis [32]. In our previous study, early TM5441 treatment remarkably decreased body weight, lipid profile, and systemic insulin resistance. Particularly, in adipose tissue, it also attenuated inflammation, increased insulin sensitivity, as well as improved mitochondrial biogenesis [26]. Therefore, it is imperative to consider the action of TM5441 in preventing adipose tissue inflammation as a great contributor in ameliorating HFD-induced NAFLD.

Accordingly, we showed hepatic lipid accumulation in HFD-fed mice progressing to hepatic inflammation as shown by massive F4/80 infiltration, a major tissue-resident macrophage in the liver [38], as well as elevated MCP-1 and NLRP3 mRNA level. Noticeably, aberrant inflammatory markers were abrogated in response to TM5441 treatment. This effect can be likely explained by a study in rat Thy-1 nephritis model treated with TM5275, an analog of TM5441. TM5275 hindered the interaction of PAI-1, identified as a chemotactic factor, with low-density lipoprotein receptor–related protein; thus, it may inhibited macrophage infiltration [21].

Albeit the notion that HFD-fed mice displayed less pronounced resemblance to pathological severity and fibrosis as seen in the liver of human NAFLD, our present study showed that 10-week-HFD might progress to liver fibrosis. Our result was then indicated the anti-fibrotic effect of TM5441, as likewise described in lung fibrosis [23] and kidney fibrosis [22] models. Collagen accumulation in the liver of HFD mice, shown by Masson's trichrome staining, was significantly reduced by TM5441. Imperatively, our study indicated that MMP-9 activation by TM544 can partially explain its anti-fibrotic effect. MMPs are a family of endopeptidases that degrade ECM with a wide range of biological activities [39]. In agreement with our result, PAI-1 deficient mice showed MMP-9 elevation leading to TIMP-1 downregulation or tPA activation that may stimulate hepatocyte growth factor, a known anti-fibrogenic protein [15, 16]. Alternatively, anti-fibrotic effect of TM5441 is likely through its direct inhibition on PAI-1 binding to hepatocyte surface receptors that might activate signaling pathway related to upregulation of TGF-β and ECM genes [40].

Aforementioned results implied that our HFD mice exhibited the spectrum of hepatic steatosis, inflammation, and eventually fibrosis. In rodents and humans with NAFLD, maladaptation of mitochondrial oxidative flux in the liver is a central feature of simple steatosis to NASH transition [41]. FFA overload on the mitochondria increases biosynthesis of toxic lipid intermediates that are potent inhibitors of insulin signaling in the liver and elicit multiple inflammatory pathways [41, 42]. This leads to higher rates of lipid peroxidation, formation of cytotoxic aldehydes, and production of pro-inflammatory cytokines, resulting in DNA damage and eventually cell death. Thus, a vicious cycle between insulin resistance and mitochondrial dysfunction is such an important target in halting the progression of NAFLD [41, 43].

In fact, both early and delayed treatment regimen showed that TM5441 elevated AMPK and PGC-1α activation, compared to HFD mice. Activation of AMPK, a master sensor to regulate metabolism, might ameliorate NAFLD via increasing hepatic insulin sensitivity, consequently suppressing hepatic de novo lipogenesis and increasing fatty acid oxidation, as well as promoting mitochondrial function in adipose tissue [44]. Interestingly, we further revealed that the upstream of AMPK activated by TM5441 treatment was CaMKKβ, not LKB1.

Furthermore, activation of PGC-1α suggested that the protection effect of TM5411 was contributed by improvement of mitochondrial biogenesis/function in NAFLD. Previous studies showed exacerbation of steatosis in mice with liver-specific deletion of PGC-1α [45]; in contrast, overexpression of hepatic PGC-1α and subsequent increases in fatty acid oxidation through elevated mitochondrial content and/or function result in reduced TG storage [46]. In our study, activation of PGC-1α was observed along with activation of its respected transcription factor, i.e. PPARα and Nrf2. Consequently, this may lead to activation of fatty acid β-oxidation enzymes, maintenance of mtDNA, expression of multiple components of the electron transport chain, as well as mitochondria biogenesis [47, 48].

However, the role of PAI-1 in regulating mitochondrial biogenesis remains controversial. The importance of PAI-1 as a therapeutic target in metabolic disorders was supported by a finding that hyperglycemia-induced mitochondrial superoxide production promoted PAI-1 expression [49]. Our previous report suggested that PAI-1 inhibition reduced adipose tissue inflammation and systemic insulin resistance through restoration of mitochondrial biogenesis [26]. Yet, another study demonstrated that cancer-associated fibroblasts overexpressing PAI-1 or PAI-2 displayed enhanced autophagy and further increases mitochondrial biogenesis in adjacent breast cancer cells [27]. Interestingly, our current results provide another evidence to support the notion that PAI-1 inhibition might reverse the deterioration of mitochondrial function.

The direct relation between PAI-1 and aberrant mitochondrial function was shown in our *in vitro* study with HepG2 cells. TNF-α stimulated-PAI-1 activity showed decreased mitochondrial biogenesis-related gene, including PGC-1α, mtDNA, NRF1, and NRF2 which were reversed by TM5441 treatment and silencing PAI-1 with siRNA. Persistently, in HepG2 cells stimulated with PAI-1 recombinant (25 & 50 nM), TM5441 pre-treatment remarkably decreased markers of fibrosis (TGF-β and fibronectin) and elevated mitochondrial biogenesis markers, including PGC-1α, mtDNA, TFAM. PGC-1α activation is such an important process that related with mitochondria numbers and the oxidative phosphorylation capacity of each mitochondrion. PGC-1α increases the expression of NRF-1 and mtTFA. The transcription of nuclear DNA-encoded respiratory chain polypeptides is increased by NRF-1, while mtTFA increases both the transcription and also the replication of mtDNA [29]. Then, *in vitro* study with PA stimulation revealed that TM5441 can directly decrease lipid accumulation and increase the intensity of mitochondria detected by BODIPY and Mitotracker^TM^, respectively. This result supported our animal study in which a decreased hepatic lipid accumulation was found along with improved mitochondrial function upon TM5441 treatment.

Corroboratively, the present study showed that TM5441 activated PGC-1α, a mitochondrial biogenesis regulator, which might 1) increase fatty acid oxidation shown by increased lipolysis-related gene, decreased lipogenesis-related gene, and reduction of hepatic steatosis; 2) enhance insulin sensitivity, presented by insulin signaling activation, plasma insulin reduction, and normalization of GTT and ITT results.

In conclusion, this recent study demonstrated that both early and delayed TM5441 treatment ameliorated hepatic steatosis in HFD-induced NAFLD. In particular, early TM5441 treatment prevented the progression of hepatic inflammation and fibrosis. Both strategies also abrogate insulin resistance and promote increases in hepatic mitochondrial biogenesis. Thus, this finding suggested that TM5441, a PAI-1 inhibitor, can be a novel therapeutic agent in NAFLD.

## MATERIALS AND METHODS

### Animal experiment

Animal studies were approved by Institutional Animal Care and Use Committee (IACUC) at Ewha Womans University (No. 2011-01-079). 10-week-old C57BL/6J male mice were housed in a room maintained at 22 ± 2°C with a 12 h dark/12 h light cycle, and fed either with normal diet (ND) or HFD (18.4% protein-derived calories, 21.3% carbohydrate-derived calories and 60% fat-derived calories, Harlan TD06414, Indianapolis, IN, USA). Control groups received 0.25% carboxymethyl cellulose by oral gavage; 20 mg/kg TM5441 was daily administered by oral gavage on HFD mice. TM5441 synthesized by Dr. Tetsuo Nakabayashi and Dr. Toshio Miyata were as described previously [18, 19].

To examine the prevention effect, 10-week-treatment of TM5441 was started along with the initiation of HFD on 10-week-old mice. Mice were sacrificed after HF feeding and treatment for 10 weeks. To examine therapeutic effect, 5-week-old mice were fed with HFD for 5 weeks, and 4-week-treatment of TM5441 was started on 10-week-old mice with glucose intolerance. Following the end of 4-week-treatment course, mice were sacrificed.

### *In vivo* insulin stimulation

For analyzing insulin signaling, mice were fasted overnight, anaesthetized and then injected via inferior vena cava with Humulin^®^ (10 U/kg, Eli Lilly, Indianapolis, IN, USA). Liver was removed 4 minutes after insulin injection.

### Glucose tolerance test (GTT) and insulin tolerance test (ITT)

After 16 h fasting, GTT was performed by orally administering 2.0 g glucose/kg body weight. Blood samples were taken from the tail vein to measure the glucose levels before and 15, 30, 60, 90 and 120 min after glucose administration. The ITT was conducted after 6 h fasting followed by an intra-peritoneal injection of 0.75 U/kg body weight Humulin (Eli Lilly). Blood glucose was measured by ACCU-Check glucose meter (Roche Diagnostics, Laval, QC, Canada).

### Measurements of metabolic parameters

Blood was centrifuged at 3,000 rpm for 15 minutes at 4°C, and plasma was collected. For plasma insulin measurements, commercial ELISA kits (R&D Systems, Minneapolis, MN, USA) were used according to the manufacturer's instruction.

### Histochemical and immunohistochemistry (IHC) analysis

Liver tissues were fixed in 4% formalin, dehydrated, and embedded in paraffin. 5-micron-section was used for subsequent staining. H&E and Masson's trichrome staining were performed to detect lipid accumulation and collagen accumulation, respectively. IHC staining was performed using immunoperoxidase procedures with a commercially available kit (Dako, Glostrup, Denmark). For detecting macrophage infiltration, anti F4/80 (1:200, Santa Cruz Biotechnology Inc., Santa Cruz, CA USA) was incubated overnight. Digital images were captured with Zeiss microscope equipped with Axio Cam HRC digital camera and Axio Cam software. For each liver section, 20 random fields were counted and mean value was used.

### Cell experiment

HepG2 cells, human hepatoma cells (ATCC, Manassas, VA, USA), were maintained in Dulbecco's Modified Eagle's Medium (DMEM, Invitrogen, Carlsbad, CA, USA), supplemented with 10% fetal bovine serum (FBS, Invitrogen), 100 U/mL penicillin, 100 μg/mL streptomycin, and 44 mM NaHCO3 at 37°C in humidified 5% CO_2_. Growth arrested and synchronized cells were exposed with 50 ng/mL tumor necrosis factor-α/TNF-α (Sigma-Aldrich, St. Louis, MO, USA) or 25 and 50 nM mouse recombinant PAI-1 (EMD Millipore, Billerica, MA, USA) in the presence and absence of 20 μM TM5441. TM5441 was added 4 hours prior to TNF-α or PAI-1 stimulation. For PAI-1 knockdown in HepG2 cells, after reaching 70–80% confluence, cells were seeded in 6-well plates with medium containing 20 nM PAI-1 siRNA (Santa Cruz, sc36179) or non-target siRNA (Bioneer, Daejeon, Republic of Korea) and LipofectaminTM RNAiMAX (Invitrogen, Carlsbad, CA, USA) according to the manufacturer's protocol and used for 24 hours transfection.

### Immunofluorescence analysis

HepG2 cells were seeded and pre-treated with either 20 or 40 μM of TM5441 for 4 hours prior to 400 μM palmitic acid (PA) stimulation for 24 hours. Neutral lipid was detected with BODIPY^TM^ 493/503 (Invitrogen, Carlsbad, CA, USA) and nucleus was subsequently stained with DAPI (Invitrogen, Carlsbad, CA, USA). With the same experimental condition, mitochondria was stained using Mitotracker^TM^ Red CMXRos (Invitrogen, Carlsbad, CA, USA). Following staining, the cells was fixed using 4% paraformaldehyde. The immunofluorescence stained cells were visualized using confocal microscopy (Carl Zeiss, Gottingen, Germany).

### Real-time quantitative reverse transcription PCR (qRT-PCR)

The expression of mRNAs was assessed by real-time qRT-PCR using SYBR Green PCR Master Mix kit (Applied Biosystems, Foster City, CA, USA) with an ABI 7300 real-time qRT-PCR thermal cycler (Applied Biosystems). The mRNA expression levels of the tested genes were normalized to 18 S and 28 S rRNA levels. Primer sequences are listed in Supplementary Table 1.

### Western blot analysis

Protein concentration in liver tissue homogenate and harvested cells was determined using the Bradford methods (Bio-Rad Laboratories, Hercules, CA, USA). Aliquots of tissue homogenates were mixed with sample buffer containing SDS and β-mercaptoethanol and heated at 95°C for 5 min. The samples were then loaded into SDS-PAGE gel and separated by electrophoresis, followed by transfer process onto a PVDF membrane (GE Healthcare BioSciences Co., Piscataway, NJ, USA). The membrane was blocked for 1 h at room temperature with 5% skimmed milk in TBS-Tween 20 buffer, followed by an overnight incubation at 4°C in a 1:1000 dilution of the indicated antibodies. The commercial antibodies used were as mentioned in Supplementary Table 2.

### Zymography

For matrix metalloproteinase (MMP) activity, zymography was performed using Novex 10% zymogram protein gels (1.0 mm, 10 well) (Life Technologies, Carlsbad, CA, USA). Liver tissue homogenized in 1 mL lysis buffer (100 mmol/L Tris, 0.5% Triton X-100 [pH 7.6]) without protease inhibitors was centrifuged (15,000 g, 15 minutes), supernatant was analyzed. Samples with equal concentrations of protein were mixed with Tris-Glycine SDS Sample Buffer (2×) (Novex). 100 μg protein was electrophoresed on zymogram gel in a non-reducing Tris-glycine SDS running buffer. Gels were washed with renaturing buffer (Novex; 25°C, 30 minutes), incubated in zymogram developing buffer (Novex; 37°C, 42 hours), stained with 0.1% Coomassie Brilliant blue R-250/50% methanol/10% acetic acid (1 hour), and finally de-stained (5% methanol/7% acetic acid).

### Statistical analysis

All results are expressed as mean ± standard error (SE). ANOVA was used to assess the differences among multiple groups. *p* value < 0.05 was considered significance.

## SUPPLEMENTARY MATERIALS FIGURES AND TABLES



## Abbreviations

ACC: acetyl-CoA carboxylase
ATGL: adipose trigyceride lipase
AMPK: AMP-activated protein kinase
BDL: bile duct ligation
CaMKKβ: Ca^2+^/calmodulin-dependent protein kinase kinase β
Cpt1α: carnitine palmitoyl CoA transferase-1 α
ECM: extracellular matrix
FFA: free fatty acid
GSK-3β: glycogen synthase kinase-3β
GTT: glucose tolerance test
H&E: hematoxylin and eosin
HFD: high-fat diet
HOMA-IR: homeostatic model assessment of insulin resistance
IHC: immunohistochemistry
ITT: insulin tolerance test
JNK: c-Jun N-terminal kinase
LKB1: liver kinase B1
MCP-1: monocyte chemoattractant protein-1
MMP: matrix metalloproteinase
mtDNA: mitochondrial DNA
NAFLD: nonalcoholic fatty liver disease
NASH: nonalcoholic steatohepatitis
ND: normal diet
NLRP3: NOD-like receptor family, pyrin domain containing 3
NRF: nuclear respiratory factor
PAI-1: plasminogen activator inhibitor-1
PGC1α: peroxisome proliferator-activated receptor γ coactivator 1α
PPAR: peroxisome proliferated-activated receptor
Scd1: Stearoyl-CoA desaturase-1
SE: standard error
SREBP-1c: sterol regulatory element binding protein 1c
TFAM: Mitochondrial transcription factor A
TG: triglyceride
TGF-β1: transforming growth factor β1
TNF-α: tumor necrosis factor-α
t-PA: tissue-type plasminogen activator
u-PA: urokinase-type plasminogen activator
