# Supplementary material for: TM5441, a plasminogen activator inhibitor-1 inhibitor, protects against high fat diet-induced non-alcoholic fatty liver disease
Source: Oncotarget. 2017 Sep 21;8(52):89746–60. doi: 10.18632/oncotarget.21120 (PMC5685706; doi:10.18632/oncotarget.21120)
Supplement: Supplementary file 1 [file oncotarget-08-89746-s001.pdf]

TM5441, a plasminogen activator inhibitor-1 inhibitor, protects against high fat diet-induced non-alcoholic fatty liver disease

SUPPLEMENTARY MATERIALS

MATERIALS AND METHODS

Animal experiment

10-week-old C57BL/6J male mice were housed in a room maintained at 22 ± 2°C with a 12 h dark/12 h light cycle, and fed either with normal diet (ND) or HFD (18.4% protein-derived calories, 21.3% carbohydrate-derived calories and 60% fat-derived calories, Harlan TD06414, Indianapolis, IN, USA). Control groups received 0.25% carboxymethyl cellulose by oral gavage; 20 mg/kg TM5441 was daily administered by oral gavage on HFD mice.

To examine therapeutic effect, 5-week-old mice were fed with HFD for 5 weeks, and 4-week-treatment of TM5441 was started on 10-week-old mice with insulin resistance. Following the end of 4-week-treatment course, mice were sacrificed.

Glucose tolerance test (GTT) and insulin tolerance test (ITT)

After 16 h fasting, GTT was performed by orally administering 2.0 g glucose/kg body weight. Blood samples were taken from the tail vein to measure the glucose levels before and 15, 30, 60, 90 and 120 min after glucose administration. The ITT was conducted after 6 h fasting followed by an intra-peritoneal injection of 0.75 U/kg body weight Humulin (Eli Lilly). Blood glucose was measured by ACCU-Check glucose meter (Roche Diagnostics, Laval, QC, Canada).

Measurements of metabolic parameters

Blood was centrifuged at 3,000 rpm for 15 minutes at 4°C, and plasma was collected. Plasma triglycerides (TG) and free fatty acid (FFA) were measured using EnzyChrom™ colorimetric assay kit (BioAssay Systems, Hayward, CA, USA). For fasted plasma PAI-1 measurement, commercial ELISA kits (R&D Systems) were used according to the manufacturer's instruction.

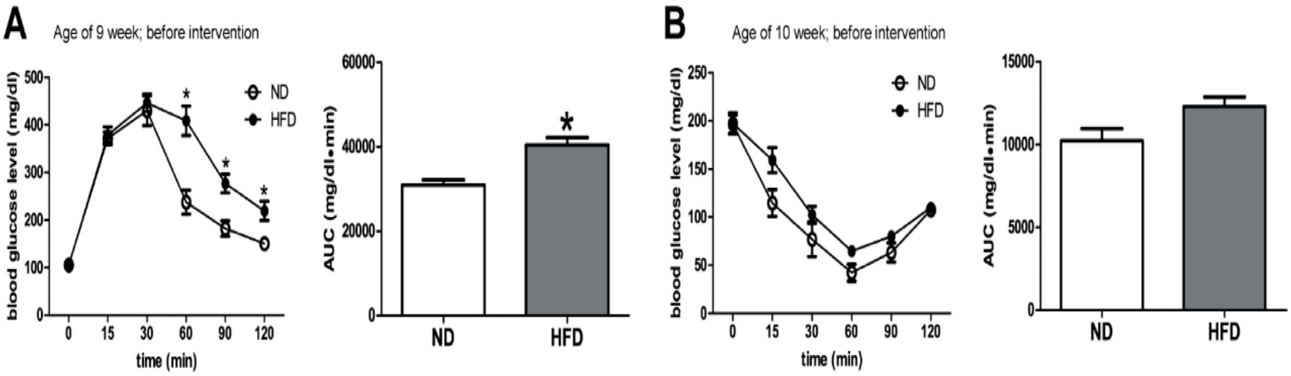

Supplementary Figure 1: Before 4-week-interventional treatment, glucose intolerance and insulin intolerance were initially confirmed in HFD mice through (**A**) GTT on 9-week-old mice and (**B**) ITT on 10-week-old mice, respectively.

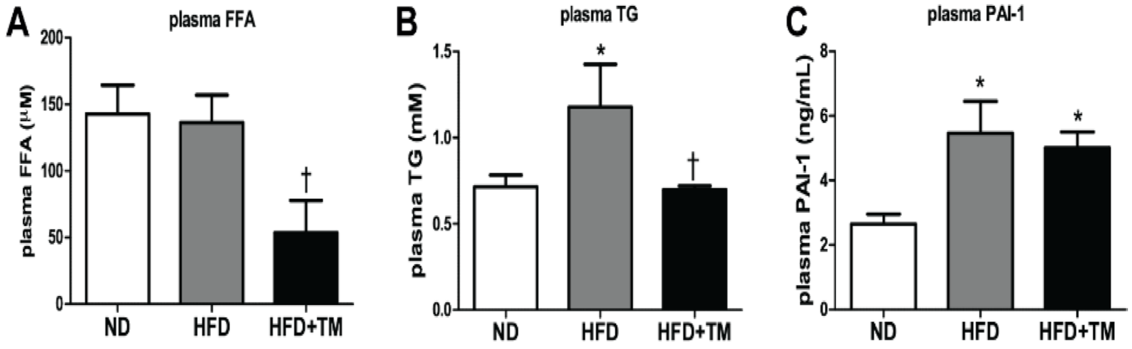

Supplementary Figure 2: Following delayed TM5441-treatment for 4 weeks, plasma (**A**) FFA, (**B**) TG, (**C**) PAI-1 were all measured in both control and treated groups.

Supplementary Table 1: The primer sequences used for real-time quantitative reverse transcription PCR (qRT-PCR)

GENE	FORWARD (5′ TO 3′)	REVERSE (5′ TO 3′)
**Mice**		
18s	CGA AAG CAT TTG CCA AGA AT	AGT CGG CAT CGT TTA TGG TC
Acc1	GCT AAA CCA GCA CTC CCG AT	GTA TCT GAG CTG ACG GAG GC
Srebp-1c	TGT GGA GCT CAA AGA CCT G	TGT GCT GCA AGA AGC GGA TG
Scd1	CAT CAT TCT CAT GGT CCT GCT	CCC ATT CGT ACA CGT CAT TCT
Cd36	CCT TAA AGG AAT CCC CGT GT	TGC ATT TGC CAA TGT CTA GC
Fasn	CCT GGA TAG CAT TCC GAA CCT	GCA CAT CTC GAA GGC TAC ACA
Cpt1α	ACC ACT GGC CGC ATG TCA AG	AGC GAG TAG CGC ATA GTC AT
Pparα	GAG AGG GCA CAC GCT AGG AA	GAA CAC CAA TGT TCG GAG CC
Pparγ	GGT GAA ACT CTG GGA GAT TC	CAA CCA TTG GGT CAG CTC TT
F4/80	CTG TAA CCG GAT GGC AAA CT	ATG GCC AAG GCA AGA CAT AC
Mcp-1	CTT CTG GGC CTG CTG TTC A	CCA GCC TAC TCA TTG GGA TCA
Tnf-α	CAT CTT CTC AAA ATT CGA GTG ACA A	TGG GAG TAG ACA AGG TAC AAC CC
Nlrp3	ATT ACC CGC CCG AGA AAG G	CAT GAG TGT GGC TAG ATC CAA G
Pai-1	AGG GCT TCA TGC CCC ACT TCT TCA	AGT AGA GGG CAT TCA CCA GCA CCA
Tgf-β1	CTT TAG GAA GGA CCT GGG TT	CAG GAG CGC ACA ATC ATG TT
Fibronectin	CGG CGT ATG CTG TCA CTG GCC G	AAG TTG AAG GCA GCC ACC TG
Collagen Iα1	GAA CAT CAC CTA CCA CTG CA	GTT GGG ATG GAG GGA GTT TA
Collagen IVα1	ATT CCT TCG TGA TGC ACA CC	GTG GGC TTC TTG AAC ATC TC
Pgc1α	TCG ATG TGT CGC CTT CTT GC	ACG AGA GCG CAT CCT TTG G
Cox4-i1	TCG ATC GTG ACT GGG TGG CCA	GCC GAG GGA GTG AGG GAG GC
mtDNA	CCA CTT CAT CTT ACC ATT TA	ATC TGC ATC TGA GTT TAA TC
**Human**		
28s	TTA AGG TAG CCA AAT GCC TCG	CCT TGG CTG TGG TTT CGC T
Pai-1	TGG AAC AAG GAT GAG ATC AG	CCG TTG AAG TAG AGG GCA TT
Pgc1α	CCA AAT GAC CCC AAG GGT TC	TAT GAG GAG GAG TGG TGG GTG
mtDNA	ACG ACC TCG ATG TTG GAT C	GCT CTG CCA TCT TAA CAA ACC
Tfam	CCT CAT CCA CCG GAG CGA TGG	ACT GAA GGG GGA GCG CAG TCG
Nrf1	CCACGTTACAGGGAGGTGAG	TGTAGCTCCCTGCTGCATCT
Nrf2	GAGAGCCCAGTCTTCATTGC	TTGGCTTCTGGACTTGGAAC

Supplementary Table 2: A list of commercial antibodies used in the study

Antibody	Manufacturer	Catalog number
F4/80	Santa Cruz	SC-71088
β-actin	Sigma-Aldrich	A5441
β -tubulin	Santa Cruz	SC-9104
ATGL	Cell Signaling	#2439
p-Akt	Cell Signaling	#9271
t-Akt	Cell Signaling	#9272
p-GSK3β	Cell Signaling	#9323
t-GSK3β	Cell Signaling	#9315
p-JNK	Cell Signaling	#9251
t-JNK	Cell Signaling	#3708
p-AMPK	Cell Signaling	#2531
t-AMPK	Cell Signaling	#2532
CaMKKβ	Santa Cruz	SC-271674
p-LKB1 (Ser428)	Cell Signaling	#3482
t-LKB1	Cell Signaling	#3047
PGC1α	Santa Cruz	SC-13067
Nrf2	Santa Cruz	SC-365949
PAI-1	Santa Cruz	SC-8979
